# Supplementary material for: Identification of photoperiod-induced specific miRNAs in the adrenal glands of Sunite sheep (Ovis aries)
Source: Front Vet Sci. 2022 Jul 22;9:888207. doi: 10.3389/fvets.2022.888207 (PMC9354845; doi:10.3389/fvets.2022.888207)
Supplement: Supplementary file 1 [file Data_Sheet_1.ZIP › Supplementary file/miRDeep2 file.zip!/miRDeep2 file/Know_Structure/LP42A2/oar-mir-16b.pdf]

miRBase precursor : oar-mir-16b
Total read count : 22012
oar-miR-16b read count : 21985
remaining reads : 27

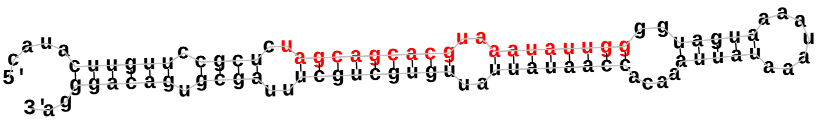

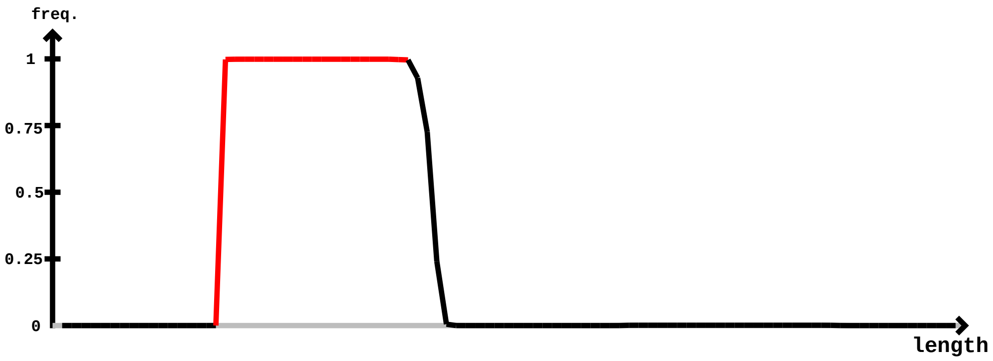

oar-miR-16b

```
5'- cauacuuguuccgcucuagcagcacguaaauauuggggguaguaaaauaaauauuaaacaccaauauuauugugcugcuuuagcgugacaggga   -3'    exp
    ....((((((.((((..(((((((((..((((((((..(((((.......)))))....))))))))..))))))))).)))).))))))..    reads      mm      sample
    ..............ucuagcagcacguaaauauugg.........................................................    1          0       seq
    ...............cuagcagcacguaaauauuggggu......................................................    1          0       seq
    ................uagcagcacguaaauau............................................................    1          0       seq
    ................uagcagcacguaaauauu...........................................................    31         0       seq
    ................uagcagcacguaaauauug..........................................................    27         0       seq
    ................uagcagcacguaaauauugg.........................................................    1488       0       seq
    ................uagcagcacguaaauauuggg........................................................    311        0       seq
    ................uagcagcacguaaauauuggA........................................................    4137       1       seq
    ................uagcagcacguaaauauugggg.......................................................    966        0       seq
    ................uagcagcacguaaauauuggAg.......................................................    9715       1       seq
    ................uagcagcacguaaauauuggggu......................................................    1887       0       seq
    ................uagcagcacguaaauauuggAgu......................................................    3285       1       seq
    ................uagcagcacguaaauauuggggua.....................................................    113        0       seq
    ................uagcagcacguaaauauugggguag....................................................    1          0       seq
    ................uagcagcacguaaauauugggguagua..................................................    1          0       seq
    .................agcagcacguaaauauuggA........................................................    6          1       seq
    .................agcagcacguaaauauuggg........................................................    1          0       seq
    .................agcagcacguaaauauugggg.......................................................    1          0       seq
    .................agcagcacguaaauauuggAg.......................................................    5          1       seq
    .................agcagcacguaaauauuggggu......................................................    4          0       seq
    .................agcagcacguaaauauuggAgu......................................................    1          1       seq
    ..................gcagcacguaaauauuggAg.......................................................    1          1       seq
    ..................gcagcacguaaauauuggAgu......................................................    1          1       seq
    ....................agcacguaaauauuggAg.......................................................    1          1       seq
    .........................................................caccaauauuauugugcugcuuu.............    2          0       seq
    ..........................................................accaauauuauugugcugcuu..............    5          0       seq
    ..........................................................accaauauuauugugcugcuuu.............    14         0       seq
    ..........................................................accaauauuauugugcugcuuua............    4          0       seq
    ...........................................................ccaauauuauugugcugcuuua............    1          0       seq
```
